# Ecological insights and societal impacts: Unveiling the relationship between outdoor sports and mental well-being among college students

**DOI:** 10.1371/journal.pone.0360170

**Published:** 2026-11-10

**Authors:** Shaohong Chen

**Affiliations:** School of Accounting, Guangzhou Huashang College, Guangdong, China; University of Naples Federico II: Universita degli Studi di Napoli Federico II, ITALY

## Abstract

This study develops and empirically validates a College Sports Ecological Risk Assessment (CSERA) model grounded in Bronfenbrenner’s multi-level social ecosystem theory to address the prevalent unidimensional limitations of conventional sports risk evaluation instruments. The proposed CSERA model encompasses four standardized risk domains—teaching risk, student personal risk, competition risk, and sports equipment risk—systematically mapped onto the micro-, meso-, and macro-system nested ecological layers of campus sports systems. Drawing on stratified probability-proportional-to-size (PPS) cluster survey data from 330 full-time undergraduates across 10 universities in Shanghai, this study adopts confirmatory factor analysis (CFA), structural equation modeling (SEM), kernel density estimation, and the Ti algorithm to test three predefined hypotheses concerning risk perception heterogeneity, the mediating role of mental well-being, and model predictive efficacy. Consistent with the results of multi-group ANOVA and bootstrap mediation analyses, teaching risk (42%) and student personal risk (35%) serve as dominant negative correlates of college outdoor sports participation. Distinct grade-based heterogeneity is identified in risk perception: freshmen demonstrate greater susceptibility to micro-system personal sports risk, whereas upper-grade students prioritize meso-system teaching risk concerns. Mental well-being exerts a statistically robust partial mediating effect on the focal association (indirect effect = 0.23, 95% CI [0.15, 0.31], 5000 bias-corrected bootstrap resamples). Cross-model MSE comparison results suggest that the CSERA model (MSE = 0.02) achieves substantially better predictive performance than hierarchical regression, risk matrix, and BP neural network models (MSE = 0.08), with its empirical stability validated via three complementary robustness tests. All observed associations are correlational given the cross-sectional self-reported design. These findings are based on undergraduates from resource-sufficient universities in Shanghai and may not be directly generalizable to institutions with limited sports infrastructure or other geographic regions. This study offers a replicable quantitative assessment tool for campus sports risk governance and provides tentative directional references for grade-differentiated physical education design that may inform campus sports safety management considerations.

## 1. Introduction

Against the backdrop of global public health resilience and sustainable development goals, regular physical activity is widely recognized as a core pathway to improving population well-being [1,2]. In higher education settings, lifestyle sports and informal physical activity have become key public health intervention carriers, supporting the physical and psychological development of young adults [3,4]. For college students in a critical developmental stage, regular exercise not only improves physical fitness but also enhances cognitive function, emotional regulation, and stress management [5–7]. Existing evidence further clarifies the psychological pathway linking physical activity to mental well-being: prior work suggests exercise is associated with better well-being via chain-mediating roles of coping styles and psychological resilience. Stable positive correlations between sports participation, mental well-being, and academic performance have been repeatedly verified [8,9].

Recent empirical data confirm a sustained rise in outdoor sports participation among young adults aged 18–25, accompanied by overall improvements in physical vitality and healthy behavioral tendencies across college populations [10–14]. From a biopsychosocial perspective, the formation of health-related behaviors is jointly shaped by individual physiological traits and environmental contexts, and the growing popularity of sports participation has correspondingly increased college students’ exposure to sports-related risks. Unlike experienced adult participants, college students are particularly vulnerable to such risks because of their inclination to explore new activities, and push physical limits without systematic preparation [15,16]. Without standardized guidance and scientific training, students may face injuries, overexertion, and other adverse health outcomes during sports participation [17]. Accordingly, growing sports participation is inevitably accompanied by increased risk exposure. Comprehensive risk-governance measures may therefore be warranted to balance participant safety and health-related benefits [18]. By promoting standardized safety practices, delivering injury prevention education, and strengthening activity supervision, higher education institutions can build a supportive environment that enables students to access sports benefits while minimizing potential hazards [19].

Current scholarly research on college sports risk management has predominantly focused on reactive response strategies, including on-campus medical rescue protocols, emergency drills, and targeted safety education campaigns [20]. However, three critical research gaps remain in building systematic assessment frameworks. First, most existing studies apply a single-dimensional analytical lens. These studies examine individual behavioral factors or facility-level conditions separately, rather than integrating multi-level interactions among individual, organizational, and societal systems. Few studies have established empirical links between sports risk perception and mental well-being outcomes [21]. Second, ecological risk assessment frameworks have been widely applied in environmental and resource management domains, but their adaptation to college sports scenarios remains insufficient, lacking a nested theoretical structure tailored to the characteristics of campus sports activities. Third, existing assessment tools generally overlook heterogeneous patterns of risk perception across academic grades, which restricts the targeted design of intervention strategies. These gaps highlight the necessity of developing a holistic, ecologically grounded risk assessment model to complement the current research system [22].

To address the aforementioned research gaps, this study develops and empirically tests the College Sports Ecological Risk Assessment (CSERA) model. This proactive, multi-level analytical framework is grounded in Bronfenbrenner’s social-ecosystem theory [23]. It represents a shift from traditional reactive risk management toward systematic, preventive risk governance. By capturing nested cross-system interactions and their influences on sports risk perception, the model offers a holistic lens for studying multifaceted sports participation risks among college students. The CSERA model integrates four core risk domains—teaching risk, student personal risk, competition risk, and sports equipment risk—across micro-, meso-, and macro-system levels, and embeds mental well-being as a key mediating mechanism explaining the linkage between risk perception and sports participation. Unlike conventional single-dimensional assessment tools such as risk matrix models and hierarchical regression analysis, the CSERA model captures cross-system dynamic interactions and incorporates the Ti algorithm, offering enhanced adaptability for college sports risk management practices.

This study pursues three interconnected research objectives:

(1) To build a multi-level CSERA model with four risk dimensions mapped onto nested ecological systems, forming a theoretical framework for college sports risk assessment.
(2) To empirically test associations among ecological risk factors, mental well-being, and outdoor sports participation using college-student questionnaire data, and to specifically test the hypothesized mediating role of mental well-being.
(3) To explore grade-specific heterogeneity in risk-perception patterns and evaluate the within-sample explanatory performance of the CSERA model against conventional assessment approaches.

Building on social ecosystem theory and prior empirical evidence [24], we propose three testable hypotheses.

H1: Higher perceived ecological sports risk is negatively associated with outdoor sports participation among college students, with teaching and students’ personal risk exerting the strongest inhibitory effects.

H2: Mental well-being partially mediates the association between ecological risk perception and outdoor sports participation, such that elevated risk perception is associated with impaired mental well-being, which in turn correlates with reduced sports participation.

H3: The salience of risk dimensions exhibits grade-level heterogeneity: freshmen perceive higher personal risk, whereas upper-grade students (including sophomores, juniors, and seniors) report higher teaching risk, corresponding to differences in sports experience and curriculum exposure.

This study contributes to the emerging field of sports ecology by integrating social ecosystem theory with mental well-being research and provides empirical evidence to inform risk-aware physical education policies and differentiated intervention strategies in higher education. The remainder of this paper is structured as follows: Application of Social Ecosystem Theory in Sports Risk Assessment elaborates on the application of social ecosystem theory in sports risk assessment and compares existing evaluation models; Materials and Methods details the construction of the CSERA model, sample design, measurement instruments, and analytical strategies; Results presents the empirical results, including grade-specific risk patterns and model performance validation; Discussion discusses the findings in relation to prior research, outlines practical implications, and acknowledges study limitations; and Conclusions concludes the paper.

## 2. Application of social ecosystem theory in sports risk assessment

The theoretical foundation for a proposed College Sports Ecological Risk Assessment (CSERA) model. First, it unpacks Bronfenbrenner’s social ecosystem theory and validates its applicability to outdoor sports risk research in colleges. Subsequently, it establishes a one-to-one mapping between the three nested ecological layers and four core sports risk domains. Subsequently, a standardized static weighting formula is developed to quantify layer-specific risk contributions. Finally, this chapter systematically critiques prevailing risk assessment paradigms, highlights the multidimensional innovations of the CSERA model, and advances three statistically testable theoretical conjectures to guide the subsequent empirical analyses in Materials and Methods.

### 2.1 Theoretical connotation and research adaptability of social ecosystem theory

Bronfenbrenner’s social ecosystem theory posits that individual sports behaviors and risk perception arise from bidirectional, dynamic interactions across three hierarchically nested environmental tiers: micro-, meso-, and macro-systems [25]. As a foundational paradigm within the emerging sports ecology subfield, this framework overcomes the single-factor analytical bias prevalent in the prior sports risk literature [26,27]. Two rationales justify its adoption for evaluating college students’ outdoor sports risks: first, risk perception is jointly shaped by individual psychology, campus teaching administration, and societal sports culture rather than isolated individual factors; second, the tiered nested structure enables a clear classification of risk sources and lays a structured basis for subsequent quantitative weight calculations.

#### 2.1.1 Micro-system.

The microsystem denotes the immediate daily interactive environment surrounding individuals, representing the innermost tier of the ecological framework [28]. For undergraduate sports participation, the core micro-level elements include personal physical fitness, exercise cognition, warm-up compliance, family sports guidance, and on-site physical education (PE) classrooms. Individual behavioral flaws are theorized to constitute the primary source of sports injuries; for instance, skipping pre-exercise warm-ups is theorized to directly increase the risk of injury, while targeted in-class risk training may help standardize movement habits. Given freshmen’s limited sports experience, the microsystem becomes the priority target for personalized risk intervention.

#### 2.1.2 Meso-system.

The mesosystem functions as an intermediate connecting layer that reflects the interactive constraints among multiple microsystems [29]. Within campus sports contexts, key mesosystem linkages include coordination between PE teaching offices and facility management departments, as well as consistent risk education inside and outside formal classes. Fragmented cross-departmental communication creates persistent safety loopholes, whereas unified home-school risk guidance can effectively raise students’ awareness of risks. All teaching and sports equipment risks defined in this study originate from meso-system operational defects, the coordination efficiency of which determines the overall campus risk governance performance.

#### 2.1.3 Macro-system.

The macro-system encompasses overarching institutional policies, cultural norms, and societal values that constrain both the micro and meso operational tiers [30]. National PE regulations, campus sports management bylaws, and public sports prevention culture collectively shape students’ subjective risk judgment and willingness to participate. Macro-system institutional frameworks provide long-term standardized safety criteria for all campus sports activities. Together with meso-system event organization mechanisms, they jointly generate cross-layer competition risk identified in this study.

Fig 1 illustrates the three-tier nested CSERA ecological framework. The inner micro-system centers on individual undergraduates, covering family and classroom scenarios; the intermediate meso-system contains campus institutional rules, management workflows, and sports facility conditions; and the outer macro-system encompasses national sports policies and broader social sports culture. Bidirectional connecting arrows reflect the mutual spillover effects across tiers: changes to any single layer alter students’ overall risk perception and their subsequent outdoor sports participation decisions.

**Fig 1:**
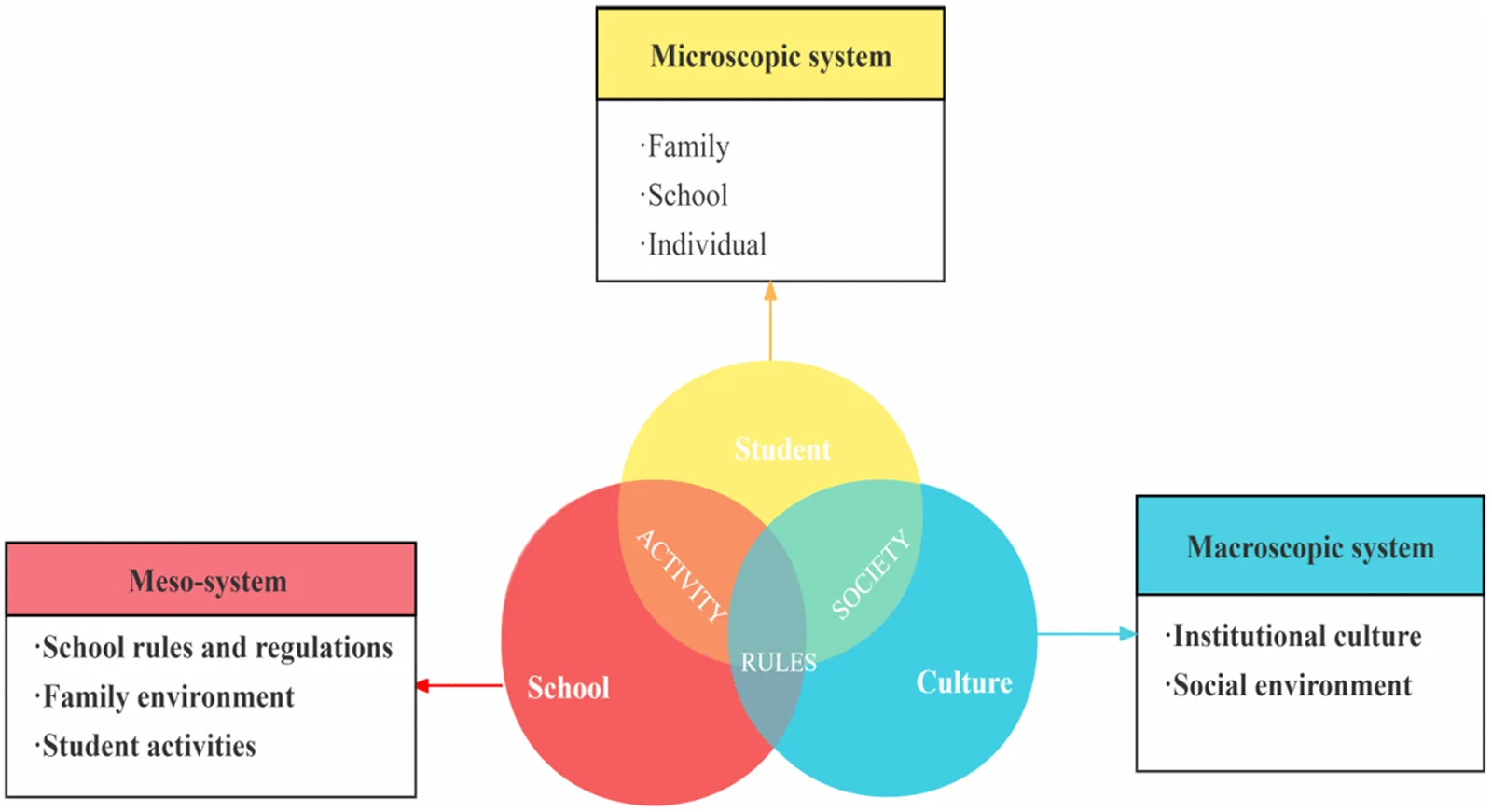
Nested ecological framework mapping four sports risk dimensions to micro-, meso-, and macro-systems for the CSERA model.

Supplementary Interpretation of Fig 1: The four core risk dimensions defined in this study precisely match the three ecological layers in the figure. Student personal risk belongs exclusively to the micro-system; teaching risk and sports equipment risk are embedded in the meso-system; competition risk spans the overlapping area of the meso-system (campus event management) and macro-system (social competitive culture), forming a cross-layer risk source that cannot be captured by single-layer analysis frameworks.

### 2.2 Ecological mapping and quantification of college sports risk factors

Two sequential tasks: first, matching the four core risk domains to the three ecological tiers via qualitative theoretical deduction; second, establishing a normalized static weighting formula to quantify each tier’s relative-risk contribution. The static weights calculated here serve as the initial input parameters for the Ti dynamic calibration algorithm detailed in Ti Dynamic Risk Weight Calibration Algorithm, forming a two-stage quantitative risk assessment workflow.

#### 2.2.1 Mapping of risk domains to ecological systems.

(1) Micro-system: Student personal risk

Rooted entirely in individual cognitive and behavioral flaws, this dimension encompasses the overestimation of physical limits, neglected warm-up procedures, and non-standard exercise movements, which act as direct triggers for sports injury incidents.

(2) Meso-system: Teaching risk & sports equipment risk

Teaching risk arises from inadequate in-class risk training, insufficient teacher on-site supervision, and mismatched PE curriculum content, which are closely tied to instructors’ professional capacity and school teaching administration [31]. Sports equipment risk stems from aging facilities, irregular maintenance cycles, and ill-fitting sports gear, which reflect operational failures in campus facility management departments.

(3) Cross meso-macro system: Competition risk

This dimension integrates the pressures from formal campus tournaments, unstandardized event planning, and distorted social competitive values [32]. Unlike the single-layer risk indicators adopted in prior evaluation tools, its cross-tier origin uniquely demonstrates the multi-source capture capacity of social ecosystem theory, representing a core theoretical novelty of the CSERA model.

#### 2.2.2 Calculation of ecological risk impact factors.

A hierarchical normalization method was adopted to compute the risk contribution weight of each ecological tier. (range: 0–1; higher values indicate stronger risk-explanatory power).


$$\begin{array}{c} \;F(x{)}_{micro}=\frac{\sum D_{x}}{\sum D_{x}+\sum \left(D_{t}+a\right)+\sum (t+b)}\;\; \end{array}$$ (1)

$$\begin{array}{c} F(x{)}_{meso}=\frac{\sum \left(D_{t}+a\right)}{\sum D_{x}+\sum \left(D_{t}+a\right)+\sum (t+b)} \end{array}$$ (2)

$$\begin{array}{c} F(x{)}_{macro}=\frac{\sum (t+b)}{\sum D_{x}+\sum \left(D_{t}+a\right)+\sum (t+b)} \end{array}$$ (3)

Variable definitions (concise list format to reduce verbose text):

(1) $F(x{)}_{micro},F(x{)}_{meso}$, $F(x{)}_{macro}$: Risk contribution weights for micro, meso, and macro systems, sum to 1 after normalization
(2) $\sum D_{x}$: Cumulative score of all micro-system (student personal risk) survey indicators
(3) $\sum \left(D_{t}+a\right)$: Cumulative score of meso-system indicators; the correction coefficient *a* = 0.15 was empirically determined using pilot questionnaire data (*n* = 30). The average systematic deviation of teaching- and facility-related risk scores within the pilot sample was approximately 15%, which justifies the use of this adjustment term to eliminate inherent systematic biases in institutional teaching and facility management evaluation indicators.
(4) ∑(t+b): Cumulative score of cross-layer competition risk indicators; social culture correction coefficient *b* = 0.10, quantifying societal competitive atmosphere’s influence on risk perception

The raw data inputs included formal questionnaire responses and official campus facility management archives. Critically, these static weights assume fixed risk proportions across all student subgroups and cannot accommodate grade-level heterogeneity. This limitation motivated the development of the adaptive Ti dynamic weight algorithm presented in Materials and Methods.

### 2.3 Comparative analysis of mainstream assessment models and CSERA rationale

Four widely adopted college sports risk evaluation paradigms were systematically benchmarked against the CSERA model to identify the structural defects of conventional tools and clarify the theoretical, mechanistic, and technical innovations of the proposed framework.

#### 2.3.1 Limitations of conventional assessment models.

(1) Hierarchical regression analysis

This tool generates static indicator weights via artificial risk grouping but cannot model cross-layer ecological interactions. Practically, it fails to distinguish risk disparities between freshmen and upper-grade students, making differentiated grade-intervention design impossible. Its single-level output only optimizes existing management schemes rather than constructing a multi-tiered prevention system.

(2) Risk matrix model

Risk grading relies on subjective dual-dimension (probability-severity) scoring, incurring high human resource costs that are incompatible with resource-limited university PE departments. Critically, this model excludes mental well-being and subjective psychological risk perception indicators from the evaluation framework.

(3) SEF (Severity-Exposure-Frequency) three-dimensional risk model

It classifies risk by severity, exposure, and frequency but lacks alignment with the three-tier nested ecological structure. It ignores students’ psychological risk perception pathways and fails to capture the mediating effect of mental well-being, central to this study’s hypotheses.

(4) SPSS standalone correlation analysis

Univariate correlation analysis was performed on single-region samples without unified ecological theoretical support, and no built-in functions were available to test grade-based risk heterogeneity.

#### 2.3.2 Methodological advantages of the CSERA model.

Against the above defects of traditional single-dimensional tools, the CSERA model, built on social ecosystem theory, has three targeted innovations.

(1) Theoretical framework innovation

Recognizes one-to-one matching between the four risk domains and the three ecological tiers. Equations (1)–(3) quantify the cross-tier risk spillover effects, thereby replacing the fragmented single-indicator evaluation with a holistic ecosystem risk measurement.

(2) Mediating mechanism innovation

This study uniquely embeds mental well-being as a core mediating variable to fill a persistent literature gap: prior risk assessment tools exclusively measure objective environmental or behavioral hazards while overlooking the psychological correlational pathway. Elevated risk perception is posited to correlate with greater sports anxiety, poorer mental well-being, and lower outdoor-sports participation. The CSERA model alone captures this full correlational pathway.

(3) Technical algorithm innovation

To overcome the fixed-weight limitations of conventional regression and matrix-based evaluation tools, this study developed a Ti dynamic weight calibration algorithm (hereafter referred to as the Ti algorithm). This algorithm adaptively adjusts risk weights across student subgroups, enabling the CSERA model to capture grade-specific heterogeneity that static models cannot accommodate.

#### 2.3.3 Theoretical preconditions for subsequent model diagnosis and robustness tests.

Based on the comparative analysis of mainstream risk evaluation tools, three testable conjectures corresponding to H1–H3 (derived from Introduction) were formulated as specific empirical expectations: superior model fit of the CSERA model, grade-specific heterogeneity in risk weight distributions, and a partial mediating pathway of mental well-being. These conjectures lay the theoretical foundation for the subsequent model diagnosis and robustness tests.

Static weights from Equations (1)–(3) and standard SEM path coefficients only reflect global fixed effects and cannot capture grade-level heterogeneity in risk perception. To address this limitation, this study developed a Ti dynamic weight calibration algorithm to realize subgroup-adaptive weight optimization, which represents a core technical innovation of the CSERA model.

A fixed random seed of 12345 was adopted for all randomization processes to ensure full reproducibility of the results, and MLR robust maximum likelihood estimation was uniformly used to mitigate minor residual nonnormality. The above theoretical deductions lay a logical foundation for the model construction and empirical testing. Materials and Methods elaborates on the research design, sampling procedure, measurement instruments, algorithm specification, and statistical analysis strategy of this study.

## 3. Materials and methods

It elaborates on the complete empirical research design based on the three research hypotheses and the multi-layer ecological theoretical framework constructed in Application of social Ecosystem Theory in Sports Risk Assessment. It systematically introduces the ethical review procedures, stratified sampling framework, sample size calculation basis, participant inclusion and exclusion criteria, questionnaire distribution and data cleaning procedures, measurement scales and psychometric testing, Ti dynamic weight calibration algorithm, unified statistical analysis procedures, and multi-dimensional robustness verification schemes. All analytical software, algorithm parameters, and statistical judgment thresholds were fully documented to guarantee the reproducibility of the entire empirical study.

### 3.1 Study design and ethical approval

A cross-sectional, one-time questionnaire design was adopted to test H1, H2, and H3, as proposed in Introduction. This design can reveal correlational associations between variables but cannot support causal inferences. All randomization operations (PPS sampling extraction, Ti algorithm weight initialization, and K-means clustering grouping) were locked with a unified fixed random seed of 12345 to eliminate random fluctuations and ensure the full reproducibility of all results. The formal research protocol obtained written ethical approval from the Ethics Committee of Guangzhou Huashang College on April 10, Approval No. HS2022−012. Electronic informed consent was mandatory before questionnaire access; participants were fully informed of the research objectives, average completion time of 8–12 minutes, full data anonymization, and voluntary quit rights. No student ID, name, campus, or other identifiable private information were collected. All raw questionnaire data were encrypted and stored on local offline servers after de-identification to comply with domestic personal data protection regulations and ensure confidentiality.

### 3.2 Sampling strategy and participant characteristics

#### 3.2.1 Sampling Procedure.

Stratified probability proportional-to-size (PPS) cluster sampling was implemented based on the official classification of 64 higher-education institutions in Shanghai released by the Shanghai Municipal Education Commission. The complete sampling workflow was divided into four sequential steps as follows:

(1) Stratification grouping

All institutions were categorized into four strata by school type: 16 comprehensive universities, 8 normal universities, 22 science and engineering universities, and 18 finance and economics universities.

(2) PPS random sampling of universities

Within each stratum, 2–3 institutions were randomly selected, with sampling weights proportional to the total undergraduate enrollment. Ten target universities were retained for the formal investigation.

(3) Cluster sampling of classes

For each selected university, 2–3 classes covering all grades (freshman to senior) and three disciplines (arts, science, and engineering) were randomly extracted, generating 50 independent class clusters for questionnaire distribution.

(4) Participant screening

All students meeting the predefined inclusion criteria in sampled classes were invited to fill out questionnaires, and no eligible participants were randomly excluded from the data collection.

#### 3.2.2 Sample Size Justification.

A target sample size of 500 was predetermined using two independent quantitative standards to ensure a sufficient statistical power.

(1) SEM empirical rule

At least 10–20 observations were required for each of the freely estimated parameters. The measurement model included 28 free parameters, requiring a minimum sample size of 280–560 participants.

(2) A priori power analysis via G*Power 3.1.9.7

A multiple regression framework with up to six predictors, *α* = 0.05, power = 0.80, and medium effect size *f²* = 0.15 [33], yielded a minimum required sample of 98. A target of 500 questionnaires was set to offset an estimated 20% invalid response rate and to support subgroup mediation and heterogeneity analyses. A 20% invalid response rate was preset in accordance with the common sampling experience of questionnaire surveys in sports psychology research.

#### 3.2.3 Eligibility criteria.

Inclusion criteria:

(1) Full-time undergraduate student status
(2) Aged 18 years or older
(3) Self-reported outdoor sports participation at least once per month over the preceding three months (including running, basketball, swimming, and cycling).

Exclusion criteria:

(1) Questionnaires with more than 10% missing item responses were excluded.
(2) Questionnaires with 15 consecutive identical Likert straight-lining responses were excluded.
(3) Questionnaires completed in less than 5 min or over 40 minutes were excluded.
(4) Participants with a history of severe psychiatric or neurological disorders were excluded.

#### 3.2.4 Data collection procedure.

A 30-participant pilot test was conducted from April 10 to April 20, 2023, to revise ambiguous item wording, unify the one-month outdoor sports recall window, and adjust the Likert scale description logic. The formal survey was launched on April 25, 2023, using the Wenjuanxing online questionnaire platform. Invitation emails with unique survey links were sent to class academic administrators for forwarding to 500 target participants. Three standardized reminder emails were sent biweekly on May 9, May 23, and June 6, 2023, to non-respondents; data collection closed on October 25, 2023, after no new valid responses were received for 14 consecutive days.

#### 3.2.5 Final sample and missing data handling.

Of the 500 distributed questionnaires, 430 raw responses were recovered (crude response rate = 86.0%). After screening against four predefined exclusion criteria, 100 invalid questionnaires were eliminated, leaving 330 valid samples (an effective response rate of 76.7%) (S1 Data). For valid samples with item missingness ≤5%, multiple imputation by chained equations (MICE) was implemented in R 4.2.1, with 20 iterative cycles. Predictive mean matching was applied to continuous variables and logistic regression to binary covariates. All study variables were integrated into the imputation model under the missing-at-random (MAR) assumption. MICE was selected over complete case analysis and simple mean substitution to reduce estimation bias in the present study.

The sample demonstrated adequate coverage of major university types and disciplinary categories in Shanghai, although the limited sample size (*n* = 330) precludes strong claims of full representativeness of the municipal undergraduate population. Demographic distributions were generally consistent with the overall Shanghai undergraduate population: chi-square tests showed no statistically significant deviations in gender (*χ²* = 1.24, *p* = 0.27) or disciplinary distribution (*χ²* = 2.18, *p* = 0.34), while the statistical power of these comparisons is relatively limited given the sample size. Samples from comprehensive universities were slightly overrepresented.

The valid sample demographics were as follows: 158 males (47.9%) and 172 females (52.1%); 85 freshmen (25.8%), 92 sophomores (27.9%), 88 juniors (26.7%), and 65 seniors (19.7%); 126 liberal arts (38.2%), 114 science (34.5%), and 90 engineering (27.3%) majors.

In this study, cumulative sample size (unit: number of participants) refers to the incremental number of participants included in model estimation, which is used to visualize the convergence trend of prediction accuracy as the sample size increases. This unified indicator was consistently adopted in Figs 6–8 to demonstrate the stability of model prediction with expanding sample size.

### 3.3 Measures and psychometric properties

#### 3.3.1 Variable operationalization.

All measurement scales were adapted from mature Chinese-language instruments and reviewed by three sports science and psychology experts to guarantee content validity and cultural adaptability [34–37].

(1) Dependent variable: Outdoor sports participation

The composite weekly exercise volume index was calculated as the product of weekly exercise frequency (days) and average single-session duration (minutes) within the past month. Standardized outdoor sports examples were listed to reduce the recall deviation.

(2) Independent variable: Ecological sports risk perception

A 12-item 5-point Likert scale (1 = strongly disagree, 5 = strongly agree) corresponding to the three-layer ecological risk framework in Application of Social Ecosystem Theory in Sports Risk Assessment, divided into four sub-dimensions, was used.

Teaching risk (three items): insufficient pre-exercise guidance, inadequate classroom supervision, and mismatched PE curriculum design. Student personal risk (3 items): overestimation of physical limits, neglected warm-up, and non-standard movement patterns. Competition risk (3 items): competitive pressure, non-standard event organization, biased competitive values (cross meso-macro system risk defined in Mapping of Risk Domains to Ecological Systems); Sports equipment risk (3 items): aging facilities, irregular maintenance, and ill-fitting sports gear.

Each risk dimension is calculated as the sum of its 3 items, with a total score ranging from 3 to 15; higher scores indicate stronger perceived risk. For the convenience of side-by-side comparison of four dimensions in kernel density plots, the horizontal axis displays the full score range covering all four dimensions.

(3) Mediator variable: mental well-being

The validated Chinese version of the Warwick-Edinburgh Mental Well-being Scale (WEMWBS) was adopted [38], containing 14 items measuring one-month emotional status and cognitive well-being; higher scores indicate superior mental well-being.

(4) Covariates

Age and sex were controlled for in all structural equation models to eliminate the confounding effects of demographics.

#### 3.3.2 Psychometric Validation.

Reliability tests adopted Cronbach’s *α* and split-half reliability coefficients. Cronbach’s *α* values were as follows: teaching risk = 0.82, student personal risk = 0.85, competition risk = 0.78, sports equipment risk = 0.79, and WEMWBS = 0.87. All subscale split-half reliabilities ranged from 0.76 to 0.83, exceeding the 0.70 acceptable threshold for internal consistency reliability.

Confirmatory factor analysis (CFA) was conducted via SPSS AMOS 26.0 to test construct validity, adopting widely recognized fit cutoffs [39]: *χ*²/*df* < 3, CFI ≥ 0.90, TLI ≥ 0.90, RMSEA≤0.08, and SRMR≤0.08.

Four-factor risk perception model: *χ²/df* = 2.28, CFI = 0.93, TLI = 0.92, RMSEA = 0.062 (90% CI [0.051, 0.07]), SRMR = 0.058.

Single-factor WEMWBS mental well-being model: *χ²* = 2.15, CFI = 0.94, TLI = 0.93, RMSEA = 0.057 (90% CI [0.046, 0.068]), SRMR = 0.052. Nested model comparison confirmed the superior fit of the four-factor risk structure relative to three-, two-, and one-factor alternative models (all *Δχ² p* < 0.001). Composite reliability (CR = 0.81–0.86) and average variance extracted (AVE = 0.52–0.58) indicated acceptable convergent validity.

Two complementary methods were used to test for common method bias.

(1) Harman single-factor test

The first unrotated component explained 28.7% of the total variance, which was below the 40% critical threshold.

(2) Unmeasured latent method factor CFA

The model fit showed no meaningful improvement (ΔCFI = 0.008, ΔRMSEA = 0.006), and all method factor loadings were non-significant, indicating negligible common method bias. Multi-group CFA tested measurement invariance across freshman and upper-grade subgroups: configural and metric invariance were supported (ΔCFI= −0.003, ΔRMSEA=−0.001, meeting ΔCFI≤−0.01, ΔRMSEA≤0.015 standards [40]). Full scalar invariance failed to hold; therefore, latent mean comparisons across grades adopted partial invariance correction in subsequent analyses.

To identify the specific sources of non-invariance, we inspected modification indices for item intercepts across the freshman and upper-grade subgroups. Based on inspection of modification indices for item intercepts, the intercepts of Item 2 from the student personal risk subscale and Item 3 from the teaching risk subscale were freely estimated across groups, while intercepts for the remaining ten risk-perception items were constrained to be equal. Full survey item texts are not included in the supporting-information data dictionary but are available from the corresponding author upon reasonable request. After freeing these two intercepts, the partial scalar invariance model achieved acceptable fit relative to the metric-invariance model (ΔCFI = −0.007, ΔRMSEA = 0.001), satisfying the partial-invariance criteria proposed in [40].

### 3.4 Ti dynamic risk weight calibration algorithm

The static normalized weight Equations (1)–(3) established in Ecological Mapping and Quantification of College Sports Risk Factors provide a theoretically driven initialization benchmark grounded in social ecosystem theory, avoiding the convergence instability and poor interpretability caused by random weight initialization. However, these static weights are calculated based on full-sample fixed proportions and cannot capture grade-level heterogeneity in risk perceptions. To address this limitation, this study developed the Ti dynamic weight calibration algorithm, a gradient descent optimization augmented with an attraction term toward the historical best performing weight vector “w” _”best”. This algorithm does not involve neural networks, reinforcement learning modules, bi level optimization, or Bayesian acquisition functions.

The Ti algorithm takes the static ecological weights calculated by Equations (1)–(3) as the initial iterative weight vectors, and iteratively optimizes risk weights on grade stratified training data to produce differentiated adaptive risk weights. Conventional static regression and raw standardized SEM path coefficients only reflect average full sample linear associations without correction for grade stratification loss. All iterative operations of the Ti algorithm were implemented in R 4.2.1 with a fixed random seed of 12345 for reproducibility. The full iterative workflow is visualized in Fig 2.

**Fig 2:**
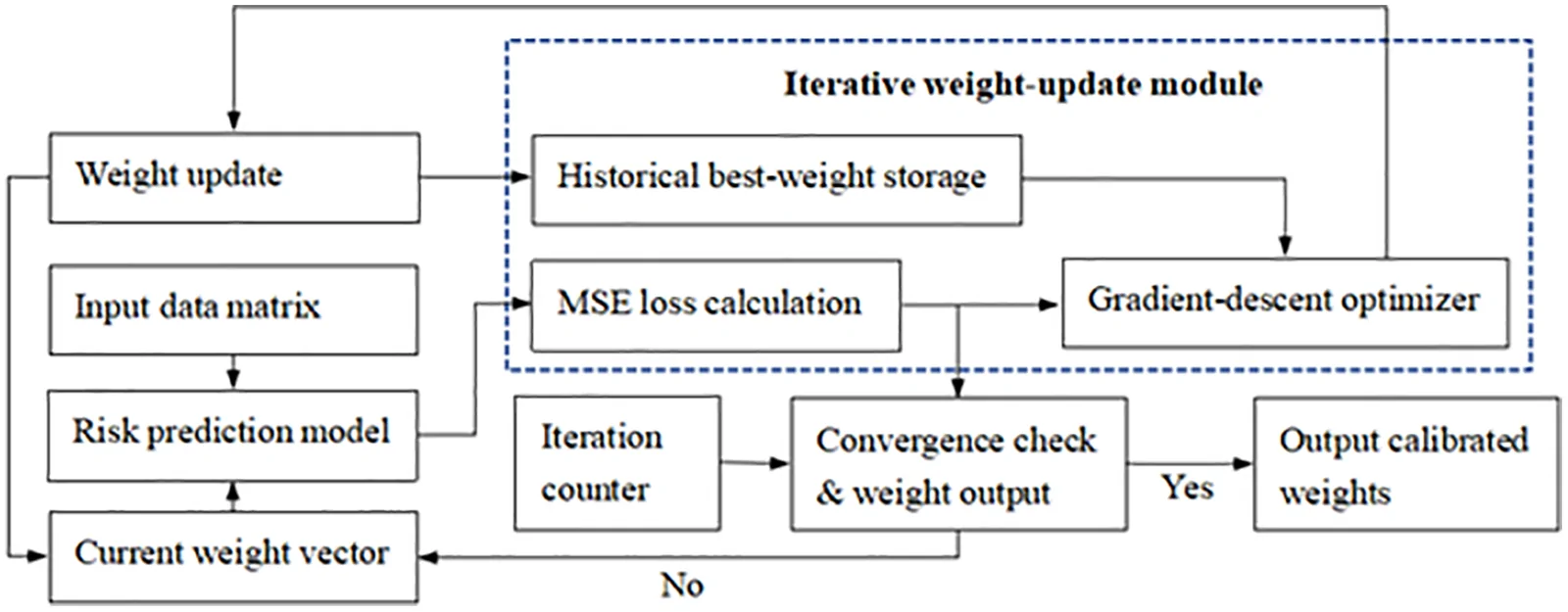
Flowchart of the Ti dynamic weight calibration algorithm based on gradient descent with an attraction term.

#### 3.4.1 Algorithm Specification.

Let $\;w^{\left(t\right)}={\left[w_{\text{1}}^{\left(t\right)},w_{\text{2}}^{\left(t\right)},w_{\text{3}}^{\left(t\right)},w_{\text{4}}^{\left(t\right)}\right]}^{T}$ represent the weight vector of the four risk dimensions (teaching, personal, competition, and equipment) at iteration *t*. The optimization objective minimizes the mean squared error (MSE) between the predicted risk score ${\hat{y}}_{i}=w^{⊤}x_{i}$ and the observed risk score $y_{i}$ for each participant i:


$$\begin{array}{c} E(w)=\frac{\text{1}}{n}\sum_{i=\text{1}}^{n}(y_{i}-w^{⊤}x_{i}{)}^{\text{2}} \end{array}$$ (4)

Weight updating rule:


$$\begin{array}{c} w^{(t+\text{1})}=w^{\left(t\right)}+\alpha \nabla \;E(w^{\left(t\right)})+\beta (w_{best}-w^{\left(t\right)}) \end{array}$$ (5)

where α = 0.01 is the learning rate, $\nabla \;E(w^{\left(t\right)})$ is the gradient of the error function with respect to the weights, *β* = 0.5 is the attraction coefficient that pulls the current solution toward the best-performing weight vector $w_{best}$ found up to iteration *t*. Termination rule: Iteration stops when the relative error change is < 10^−4^ or a maximum of 500 iterations are reached.

Hyperparameter tuning for learning rate *α* and attraction coefficient *β* was implemented via a restricted grid-search strategy. The search space was predefined: *α* ∈ {0.001, 0.005, 0.01, 0.05, 0.1}, *β* ∈ {0.1, 0.3, 0.5, 0.7, 0.9}. To avoid data leakage and mitigate the risk of sample-specific overfitting, stratified held-out validation was adopted in place of k-fold cross-validation given the moderate total sample size (n = 330). The full sample (n = 330) was split into a training set (70%, n = 231, stratified by freshman/ upper-grade subgroups) for weight iteration and a held-out validation set (30%, n = 99) exclusively for hyperparameter selection. We selected the optimal hyperparameter pair (*α* = 0.01, *β* = 0.5) that minimized mean squared error on the held-out validation dataset, rather than optimizing performance on the full analytical sample.

Multiple safeguards were applied to mitigate overfitting risks, including strict pre-specified convergence termination criteria. Additional bootstrap-based noise-perturbation tests and independent subgroup re-estimation are reported in Three Complementary Robustness Verification Schemes as post-hoc robustness checks. Considering *n* = 330 and the absence of k-fold cross-validation, sample-specific overfitting remains a non-negligible risk. Consequently, Ti-calibrated weights and hyper-parameters should be interpreted cautiously; they are optimized for this study’s sample and may not generalize directly to independent student populations and require further out-of-sample validation.

Prior to formal empirical calculation, pre-stability tests were performed including 10 independent runs with random initial weights and 100 bootstrap samples injected with ±10% random noise.

The input data matrix X, y and the current weight vector $w^{\left(t\right)}$ are used to compute the predicted risk score ${\hat{y}}_{i}=w^{⊤}x_{i}$. The MSE loss $E(w)=\frac{\text{1}}{n}\sum_{i=\text{1}}^{n}(y_{i}-{\hat{y}}_{i}{)}^{\text{2}}$ (Equation (4)) is evaluated and passed to the gradient-descent optimizer with attraction term. The weight update follows $w^{(t+\text{1})}=w^{\left(t\right)}+\alpha \nabla \;E(w^{\left(t\right)})+\beta (w_{best}-w^{\left(t\right)})$ (Equation (5)), where *α* = 0.01 is the learning rate, *β* = 0.5 is the attraction coefficient, and $w_{best}$ is the best-performing weight vector stored during iteration.The updated weights are stored as the new current weight vector and used to update $w_{best}$. The convergence check uses the MSE loss and the iteration counter; if convergence is not reached, the algorithm returns to the current weight vector for the next iteration, otherwise the calibrated weights are output for subsequent analyses.

### 3.5 Unified statistical analysis strategy

All statistical operations were performed using SPSS 26.0, Mplus 8.3, and R 4.2.1. The key R packages and versions used were lavaan 0.6–15 (CFA/SEM), mice 3.14.0 (multiple imputation), psych 2.2.9 (reliability), and tidyverse 2.0.0 (data processing). Kernel density estimation uniformly adopted a fixed bandwidth of 0.8 for all group and model comparison plots to maintain consistent visual standards.

#### 3.5.1 Core statistical premises.

The structural model is built on four basic statistical premises that are verified before the formal estimation.

Linear associations were observed between latent risk indicators and outdoor sports participation.

Residual normality was systematically diagnosed. Univariate Shapiro-Wilk tests on standardized residuals of all latent variables indicated approximate univariate normality, with skewness values within ±0.5 and kurtosis values below 1.0. Multivariate Mardia’s test detected only a minor deviation in kurtosis. Given this mild departure rather than severe non-normality, the MLR (maximum likelihood estimation with robust standard errors) estimator was adopted to adjust standard errors and ensure estimation reliability.

No severe multicollinearity exists among the variables, with VIF < 5 set as the diagnostic threshold.

Partial scalar measurement invariance was held across grade groups to support valid comparisons of latent mean values. The variance inflation factor (VIF) for all predictors ranged from 1.23 to 2.17, which was well below the conservative threshold of 5, indicating no substantial multicollinearity in the dataset.

#### 3.5.2 Four-step analytical pipeline.

The full analytical pipeline was designed to sequentially test H1, H2, and H3, with all judgment thresholds pre-defined to enhance analytical transparency.

(1) Descriptive statistical inspection

Calculate mean, standard deviation, skewness, and kurtosis for all latent variables; conduct Shapiro-Wilk test for univariate normality and Levene’s test for variance homogeneity across grades. One-way ANOVA with LSD post-hoc tests examines grade differences in risk perception, with partial *η*² reported as the standardized effect size per Cohen’s benchmark [41].

(2) Two-step structural equation modeling

Step 1: CFA measurement model validation; Step 2: Structural model estimation with MLR robust maximum likelihood. Age and sex were included as covariates in the analysis. For the mediation effect test (Hypothesis 2), bias-corrected bootstrap sampling with 5000 replications was used. A mediation pathway was defined as statistically validated when the 95% confidence interval did not contain zero (*α* = 0.05).

(3) Multi-group invariance testing

Multi-group CFA was conducted to compare risk factor loadings and latent means between freshmen and upper-grade students to verify Hypothesis 3 (grade heterogeneity). Partial scalar invariance correction was applied when full invariance failed. Partial *η*² served as the standardized effect size indicator, and a *p-*value < 0.01 was set as the threshold for statistically significant grade disparities in the risk weight distribution.

(4) Cross-model quantitative comparison

To verify the comprehensive performance advantage of the CSERA model (Hypothesis 1), five mainstream evaluation tools and a null baseline model were set as control benchmarks: hierarchical regression, risk matrix, SEF model, SPSS standalone correlation analysis, BP neural network, and naive mean prediction baseline. Two unified evaluation metrics were selected: mean squared error (MSE) as the point estimation indicator and kernel density concentration to reflect overall explanatory power. Model fit was evaluated against widely recognized cutoff criteria, and paired-sample *t*-tests were used to compare MSE differences across models, with a significance level of *p* < 0.05.

### 3.6 Three complementary robustness verification schemes

Three mutually complementary robustness checks were designed to verify the stability of the model outputs and eliminate potential sampling deviations, data noise and outlier interference.

(1) *K*-means clustering subgroup reestimation

The elbow method combined with silhouette coefficients was used to determine the optimal cluster number K = 3. The CFA and SEM models were re-estimated for each subgroup, and the model was deemed robust if all subgroups met the standardized fit thresholds.

(2) Input data noise perturbation test

±10% random noise was injected into the original questionnaire indicators before rerunning the Ti algorithm. The unchanged ranking of the risk dimension weights was interpreted as evidence of a stable dimension contribution order.

(3) Outlier sensitivity elimination test

Samples with studentized residuals exceeding ±2.5 or Cook’s distance > 1 were excluded, and the core structural model was refitted. Mediation results were considered robust if the fluctuation range of the core indirect effect coefficients remained within a 10% threshold.

## 4. Results

The empirical results corresponding to the three hypotheses was reported, including grade heterogeneity of risk perception, model fit and comparative performance, robustness verification, and mediating effect of mental well-being.

### 4.1 Grade heterogeneity of college students’ sports risk perception

A one-way ANOVA was conducted to test Hypothesis 3 regarding grade differences in risk perception.

#### 4.1.1 Risk perception distribution of freshmen (*n*=85).

Fig 3 presents the kernel density curves with a fixed bandwidth of 0.8, as standardized in Unified Statistical Analysis Strategy. Student personal risk showed the highest density peak at a total item score of approximately 14 (0.16, partial *η*² = 0.062, *p* < 0.01), which represents a medium effect size according to Cohen’s criteria. Freshmen had a significantly higher average personal risk score (*M* = 3.82, *SD* = 0.65) than upper-grade students (mean difference = 0.87, 95% CI [0.62, 1.12]). Teaching and competition risks ranked second and third in distribution concentration, whereas sports equipment risk exhibited the weakest density distribution. This pattern provides preliminary empirical support for Hypothesis 3 within the Shanghai-based sample.

**Fig 3:**
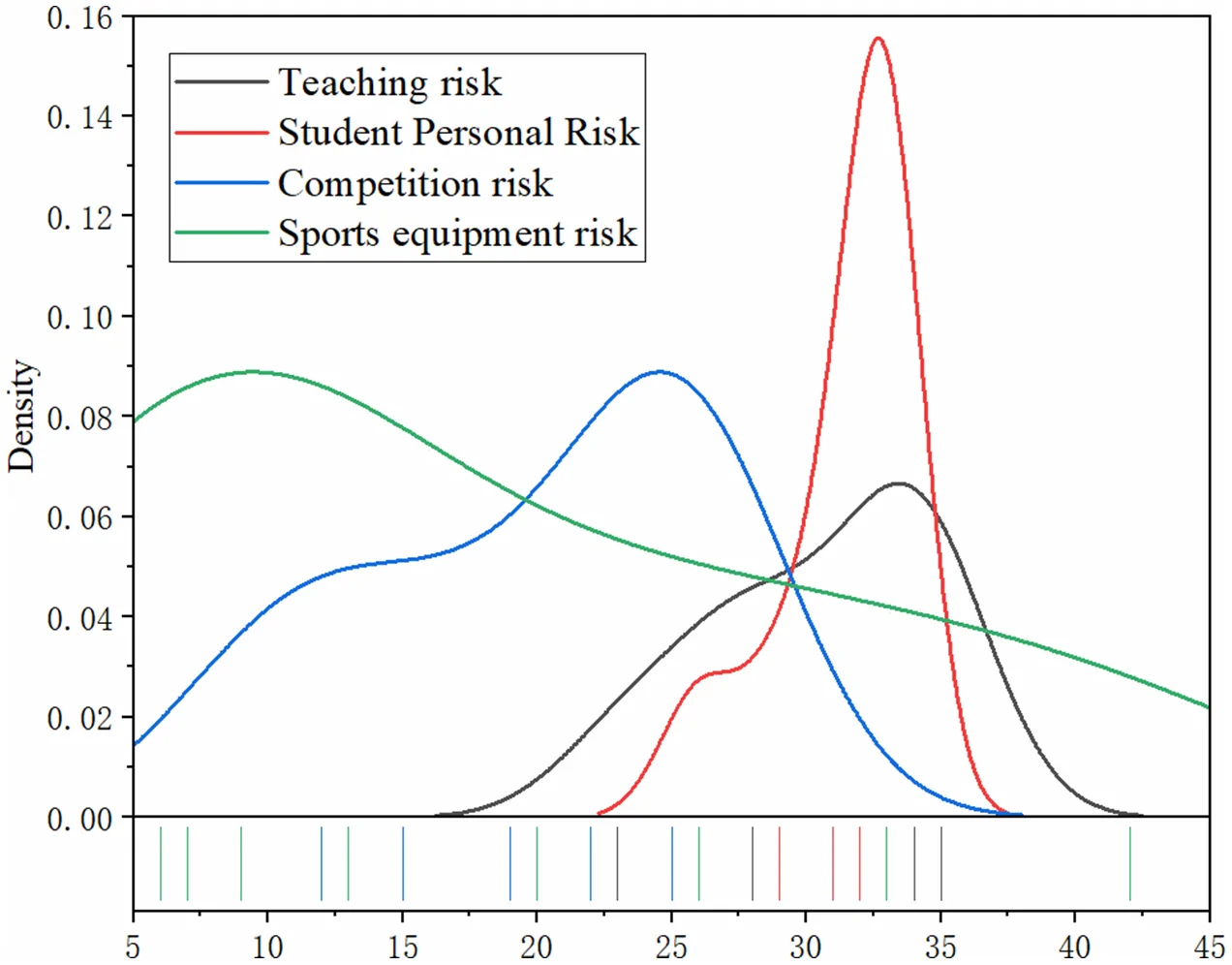
Kernel density distribution of sports risk perception among freshmen. Note: X-axis = total item score of each risk dimension (3–15 points, sum of 3 items per dimension). The 0–45 axis range covers the full score span of all four dimensions for side-by-side comparison. Y-axis = kernel density; *n* = 85, bandwidth = 0.8, partial *η*² = 0.062, *p* < 0.01.

#### 4.1.2 Risk perception distribution of upper-grade students (*n*=245).

As shown in Fig 4, the relative density order of risk dimensions reverses among upper-grade students (including sophomores, juniors, and seniors). The peak density of student personal risk declines to 0.13 at a total item score of approximately 12 (*p* < 0.05), whereas the density of teaching risk rises to 0.12 at a total item score of approximately 14 (partial *η*² = 0.055, *p* < 0.01) and becomes the primary risk factor. Upper-grade students score significantly higher on teaching risk (*M* = 3.56, *SD* = 0.68) than freshmen (mean difference = 0.44, 95% CI [0.25, 0.63]). The density of competition risk and sports equipment increased slightly. This outcome further supports the grade heterogeneity pattern.

**Fig 4:**
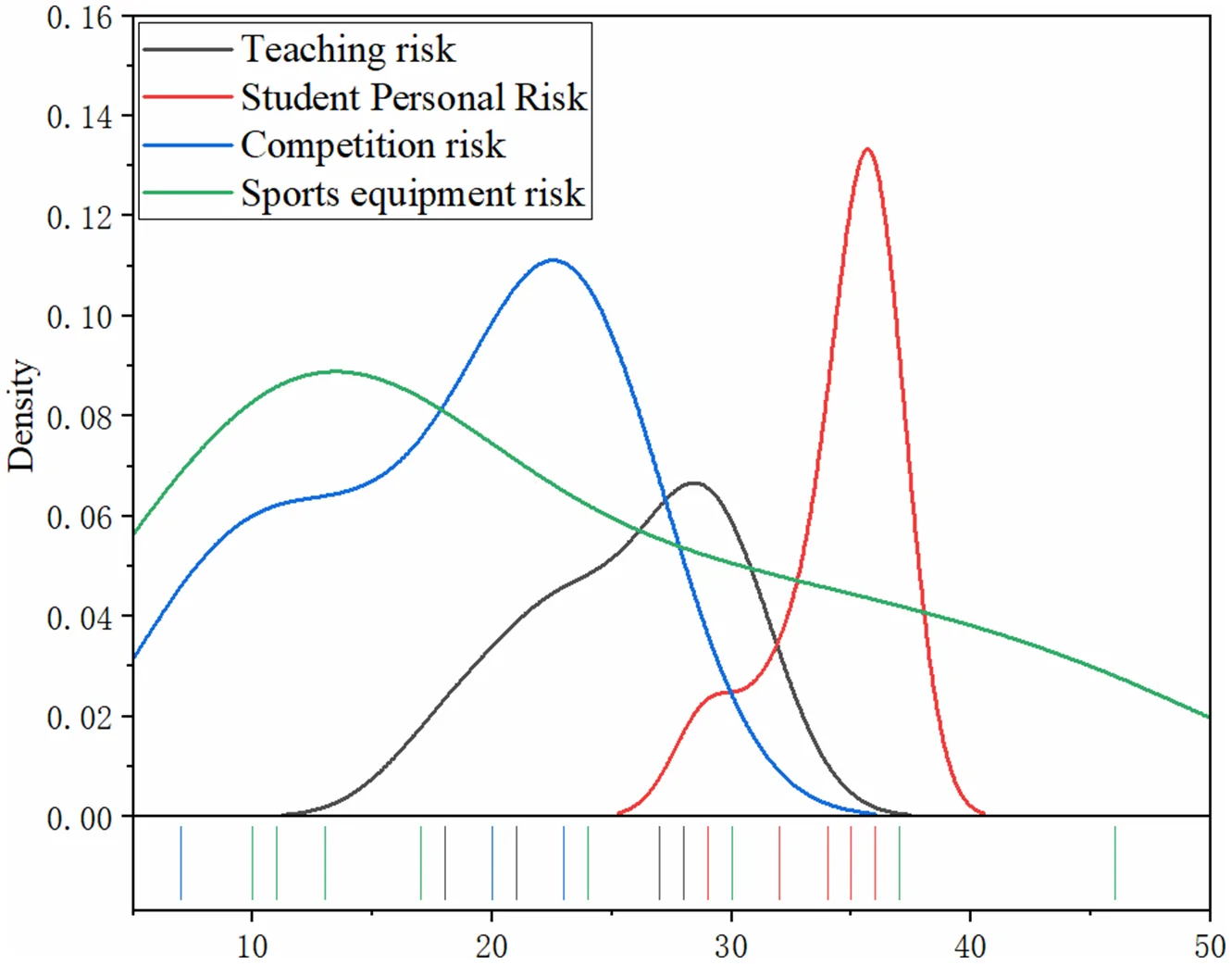
Kernel density distribution of sports risk perception among upper-grade students. Note: X-axis = total item score of each risk dimension (3–15 points, sum of 3 items per dimension). The 0–50 axis range covers the full score span of all four dimensions for side-by-side comparison. Y-axis = kernel density; bandwidth = 0.8, partial *η*² = 0.055, *p* < 0.01.

#### 4.1.3 Comprehensive ANOVA subgroup test results.

One-way ANOVA detected significant overall inter-group differences across four risk dimensions (*F* = 6.28, *p* < 0.01, partial *η²* = 0.058). Subgroup comparisons were simplified into freshman and upper-grade groups for concise heterogeneity analysis, with no significant differences detected among sophomores, juniors, and seniors. Post-hoc LSD tests identified significant gaps in student personal risk and teaching risk between freshmen and upper-grade cohorts, while competition risk and sports equipment risk showed no statistically meaningful grade differences (*p* > 0.05).

Prior to ANOVA implementation, Shapiro–Wilk normality tests (*W* = 0.97–0.99, all *p* > 0.05) and Levene’s homogeneity of variance tests (*F* = 1.12–1.89, all *p* > 0.05) were conducted in accordance with Unified Statistical Analysis Strategy; all subgroup data satisfied normality and homoscedasticity assumptions.

#### 4.1.4 Cross-model quantitative performance comparison (Test of Hypothesis 1).

To evaluate the predictive superiority of the CSERA model, this analysis follows the cross-model comparison framework designed in Unified Statistical Analysis Strategy, adopting MSE and kernel density concentration as unified evaluation indicators to contrast CSERA with hierarchical regression, risk matrix, SEF model, SPSS standalone correlation analysis, and BP neural network models.

Fig 5 presents the kernel density curves reflecting the fitting concentration of all competing models. The risk matrix reached a peak density of 0.07, followed by the BP neural network (0.06) and hierarchical regression (0.05), while the naive baseline prediction showed the lowest density concentration at 0.03.

**Fig 5:**
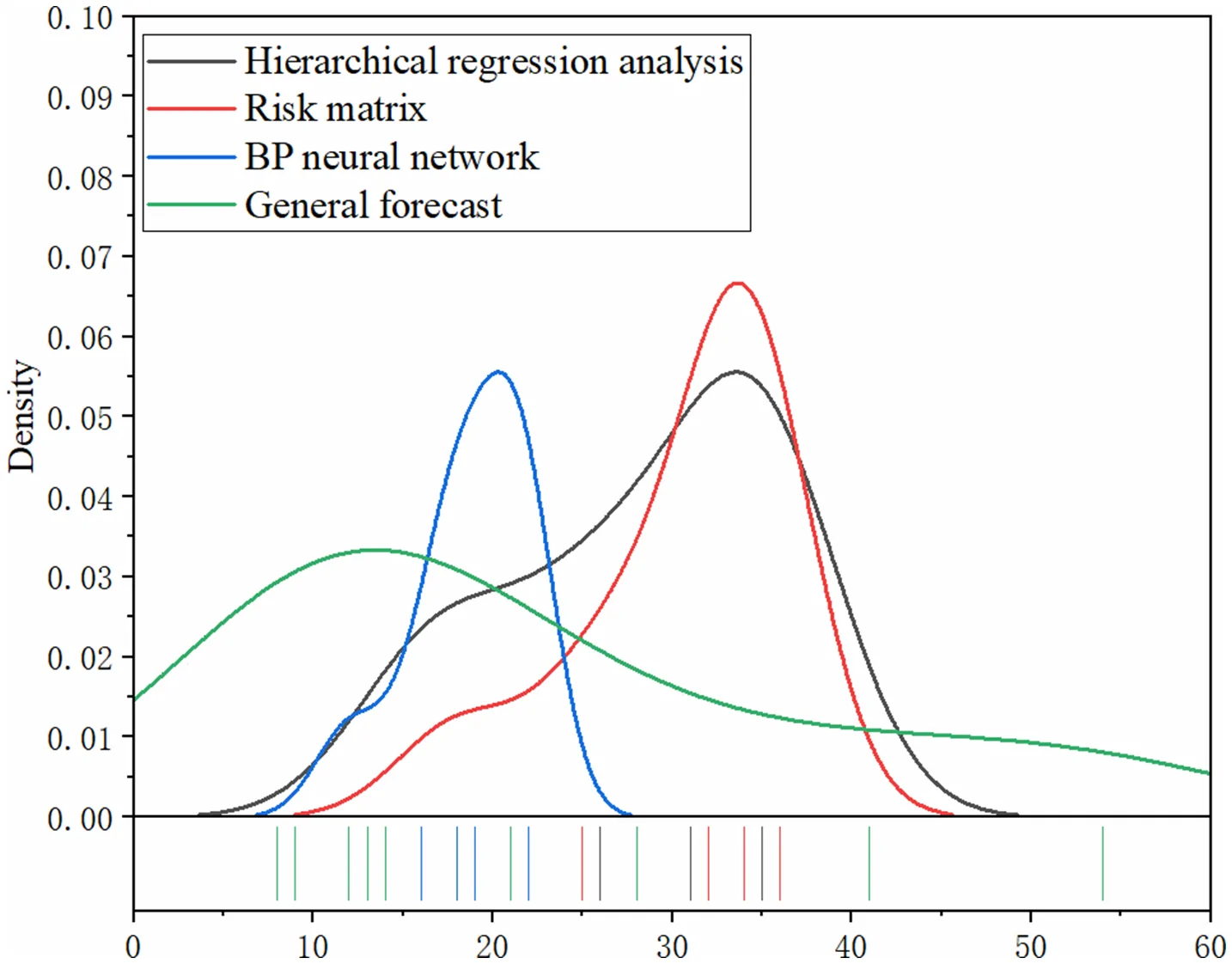
Kernel density distribution of fitting performance across alternative risk assessment models. Note: X-axis = comprehensive model fitting score (0–60, calculated based on MSE and kernel density concentration; higher values indicate better prediction performance). Y-axis = kernel density.

Fig 6 further quantifies the gap in prediction error as the cumulative sample size increases: the simplified basic ecological model (without mental well-being mediation) has an MSE of 0.08 (*p* < 0.01), whereas the full CSERA model achieves a minimal MSE of 0.02, with statistically significant performance improvements relative to all alternative models (all *p* < 0.05). The curve labelled observed actual risk values represents the real measured sports risk scores collected from participants. All pairwise MSE values and corresponding significance levels are reported in the text and figure annotations.

**Fig 6:**
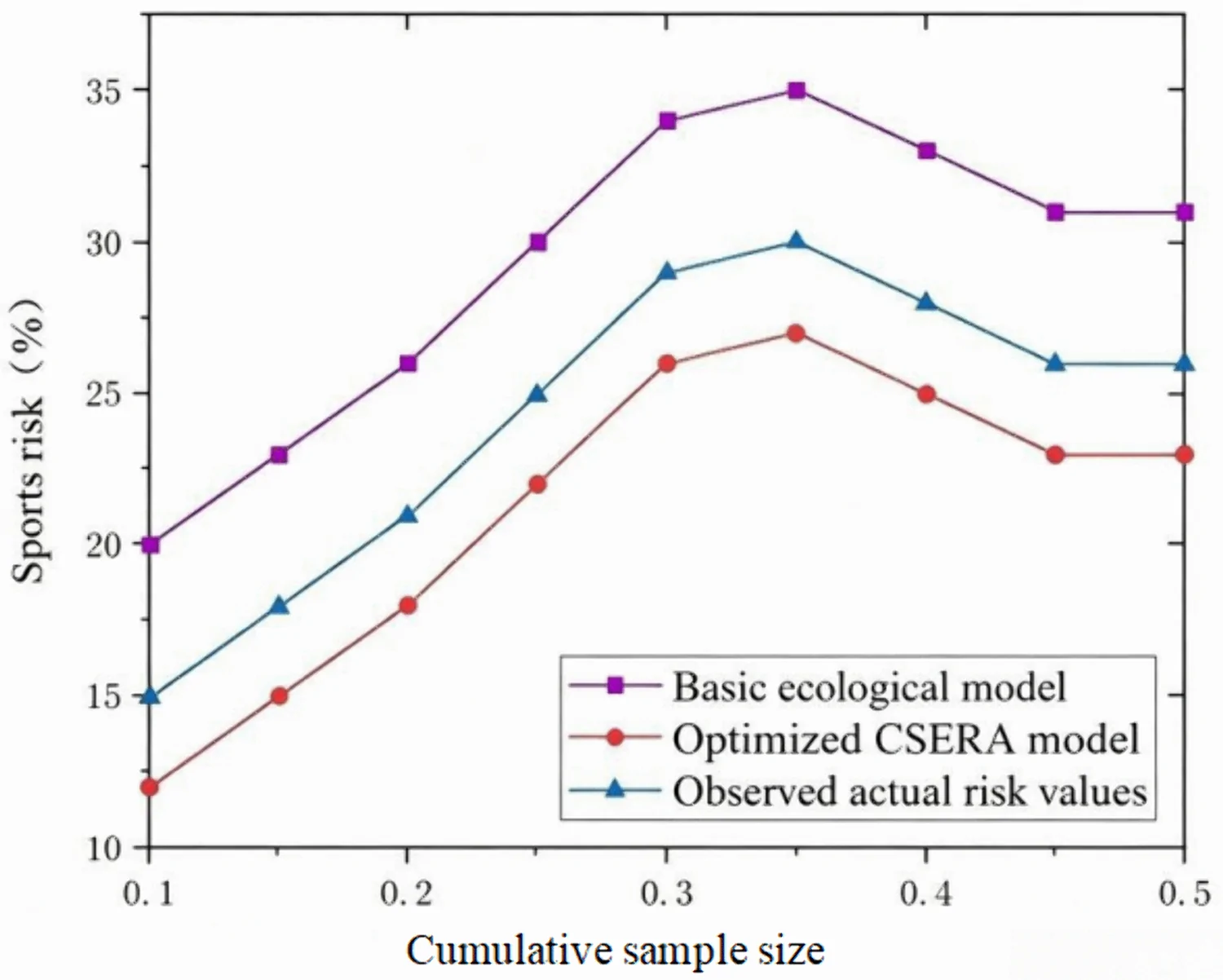
Comparative analysis of explanatory performance across ecological risk models. Note: X-axis = cumulative sample size. Each 0.1 unit on the horizontal axis corresponds to 100 participants; the valid analytical range is 100–330 participants (0.1–0.33 axis units). Y-axis = sports risk percentage; MSE(basic model)=0.08; MSE(CSERA)=0.02; all pairwise comparisons yield *p* < 0.05.

The superior fitting capacity of CSERA may be attributed to the three structural innovations summarized in Methodological Advantages of the CSERA Model three-layer nested ecological matching, embedded mental well-being mediating mechanism, and Ti algorithm, which are absent from traditional static single-layer evaluation tools.

### 4.2 Robustness test outcomes for the CSERA model

Three complementary robustness tests were conducted to verify the stability of the model’s outputs.

#### 4.2.1 *K*-Means clustering subgroup re-estimation.

*K*-means clustering was performed using the R stats package following the specifications in Three Complementary Robustness Verification Schemes. The elbow method combined with silhouette coefficient screening determined the optimal cluster number K = 3: high-risk group (*n* = 78, 23.6%), medium-risk group (*n* = 165, 50.0%), and low-risk group (*n* = 87, 26.4%). The silhouette coefficient was 0.52, which met the standard threshold of above 0.5. Separate CFA and SEM re-estimations were performed for each subgroup, and all clusters satisfied the unified fit criteria *χ²/df* < 3, CFI ≥ 0.90, and RMSEA≤0.08. Consistent acceptable fitting performance across three risk subgroups eliminated sampling segmentation bias and indicated the stability of the CSERA model.

#### 4.2.2 Correlation between model estimates and cumulative sample size.

Fig 7 plots the association between two sets of CSERA risk prediction outputs and cumulative sample size ranging from 100 to 330 participants. The static risk values derived from fixed ecological weighting formulas (green curve) and the optimized risk scores calibrated via the Ti algorithm (red curve) both show a strong positive linear correlation with cumulative sample size (*r* = 0.89, *p* < 0.001). The two prediction curves rise rapidly before the 300-participant threshold and subsequently enter a gentle stable plateau, which aligns perfectly with the two-stage static-Ti dynamic risk assessment workflow established in Ecological Mapping and Quantification of College Sports Risk Factors” and Ti Dynamic Risk Weight Calibration Algorithm.

**Fig 7:**
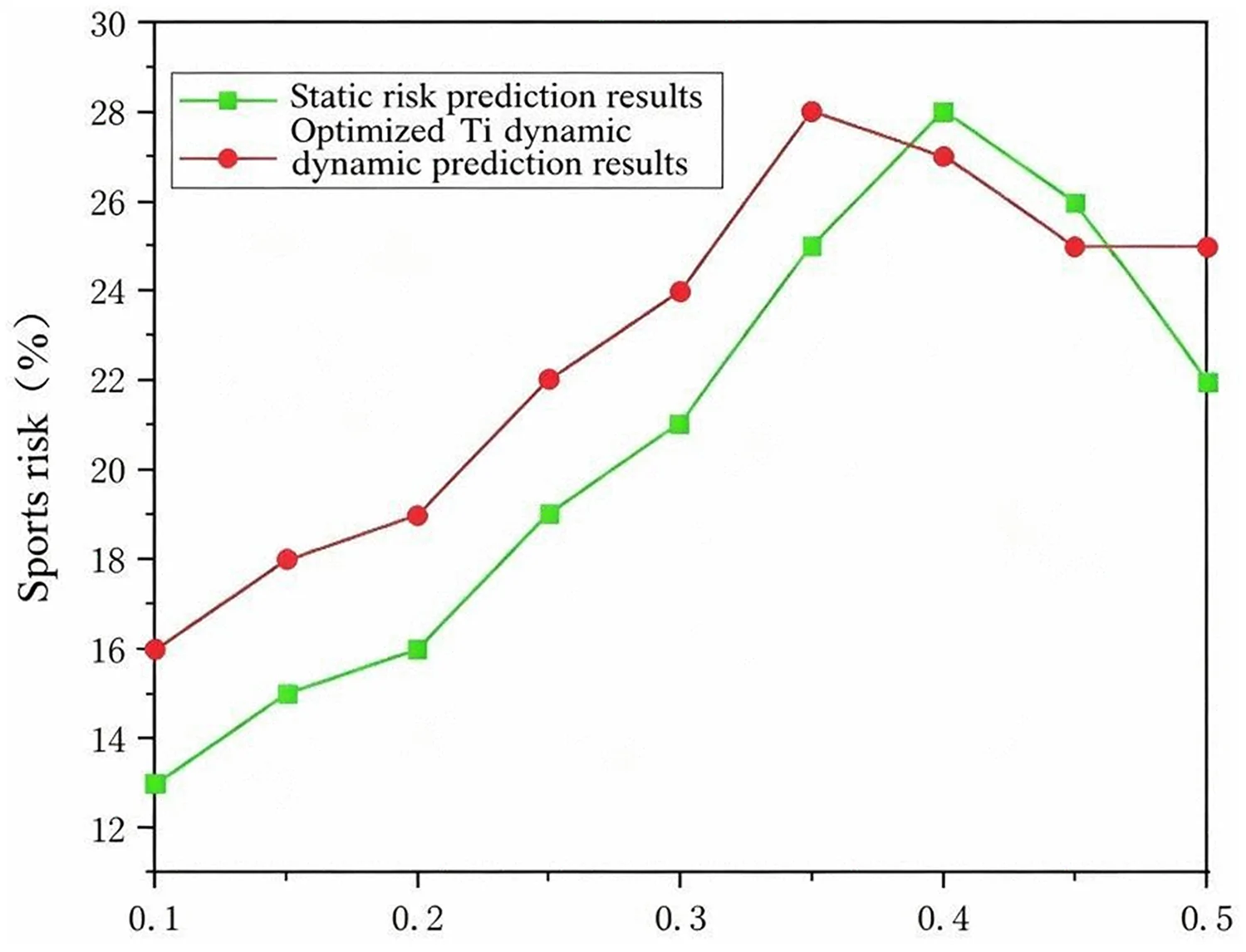
Comparison of static and Ti dynamic risk prediction results of the CSERA model. Note: X-axis = cumulative sample size. Each 0.1 unit on the horizontal axis corresponds to 100 participants; the valid analytical range is 100–330 participants (0.1–0.33 axis units). Y-axis = sports risk (%). The green curve corresponds to static risk prediction results based on fixed ecological weighting formulas, and the red curve denotes optimized Ti dynamic prediction results calibrated by the Ti algorithm.

#### 4.2.3 Comparative adaptability of classic assessment frameworks.

Fig 8 shows the risk output curves of the CFA, SEF, and SPSS standalone correlation models across different cumulative sample size ranges. In the range of 200–330 participants, the SPSS model showed a risk ratio of 30%–35%, the SEF model exhibited 15%–25%, and the CFA model remained below 15%. These patterns were consistent with the model comparison results presented in 4.1.4 Cross-model Quantitative Performance Comparison, test of Hypothesis 1”.

**Fig 8:**
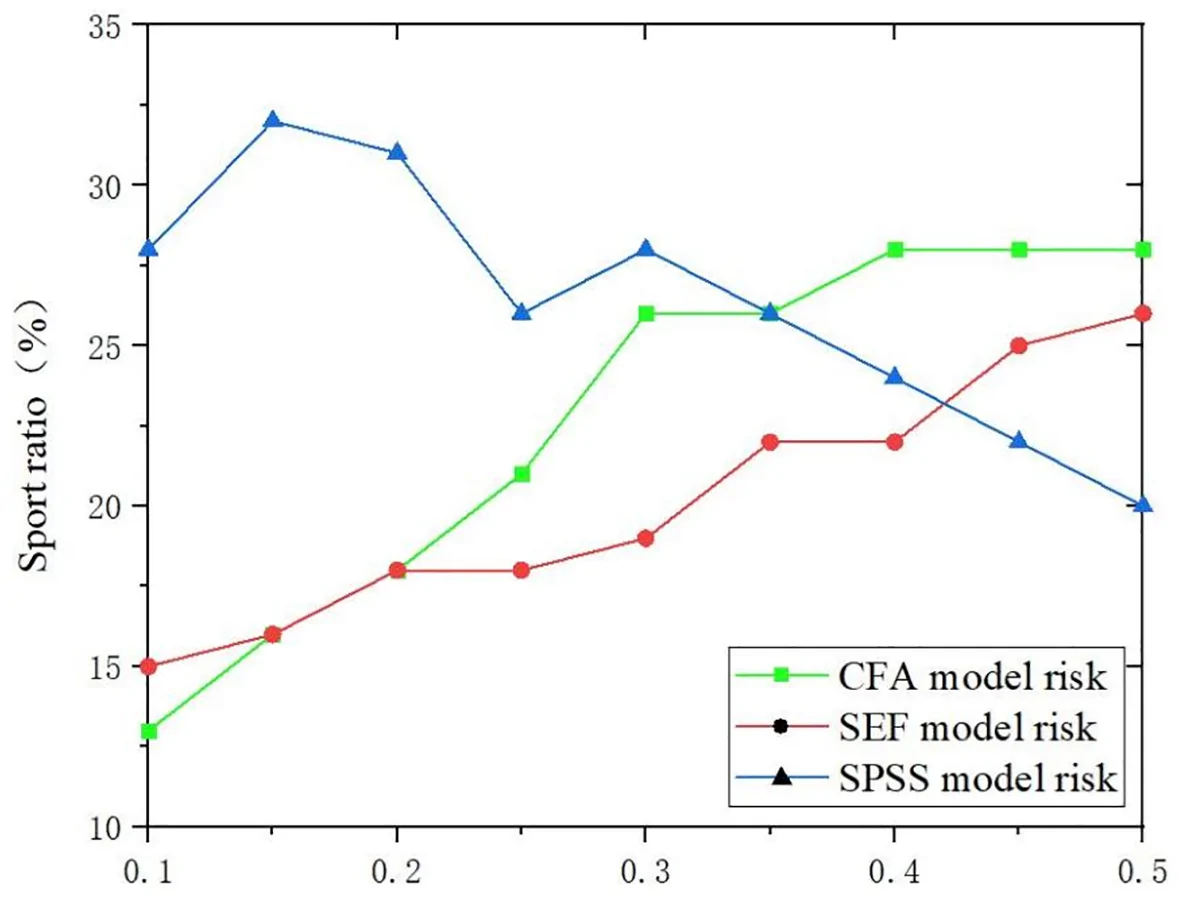
Association between cumulative sample size and sports risk ratio across classic assessment frameworks. Note: X-axis = cumulative sample size. Each 0.1 unit on the horizontal axis corresponds to 100 participants; the valid analytical range is 100–330 participants (0.1–0.33 axis units). Y-axis = sports risk ratio (%).

#### 4.2.4 Sensitivity to noise perturbation and outlier exclusion.

The Ti algorithm was rerun with 100 bootstrap samples containing ±10% random noise added to the input data. The weight ranking of the four risk dimensions remained unchanged across all perturbed samples, with coefficients of variation below 5% for each weight estimation.

The SEM model was re-estimated after excluding cases with studentized residuals exceeding ±2.5 or Cook‘s distance>1 (12 cases, 3.6% of the sample). The indirect effect coefficients remained stable (student personal risk → mental well-being →participation: 0.22 vs. 0.23; teaching risk → mental well-being → participation: 0.17 vs. 0.18), with changes well within the pre-specified 10% fluctuation threshold. These results confirm that the mediation findings are not driven by extreme observations.

### 4.3 Risk weight decomposition and indirect effect test for mental well-being (Test of Hypothesis 2)

#### 4.3.1 Calculation of dimension risk contribution weights.

The initial theoretical static weights derived from Equations (1)–(3) were 38% for teaching risk, 32% for student personal risk, 15% for sports equipment risk, and 15% for competition risk. After iterative calibration via the Ti algorithm, the converged adaptive weights were decomposed as follows: teaching risk (42%), student personal risk (35%), sports equipment risk (13%), and competition risk (10%). The subgroup-adjusted weights overcome the fixed-proportion limitation of traditional static SEM coefficients and provide a quantitative basis that may inform differentiated risk assessment and targeted intervention-related design considerations for freshmen and upper-grade students.

#### 4.3.2 Partial mediation effect of mental well-being.

This indirect effect test strictly follows the bias-corrected bootstrap scheme (5000 resamples, *α* = 0.05) specified in Unified Statistical Analysis Strategy. Two core indirect pathways were found to be statistically significant.

(1) Student personal risk → Mental well-being → Outdoor sports participation: indirect effect = 0.23, 95% CI [0.15, 0.31], CI excludes zero (*p* < 0.01).
(2) Teaching risk → Mental well-being → Outdoor sports participation: indirect effect = 0.18, 95% CI [0.10, 0.26], CI excludes zero (*p* < 0.01).

The significant indirect effects provide empirical support for Hypothesis 2. All structural models adopted MLR robust maximum likelihood estimation to offset minor residual non-normality, as predefined in Materials and Methods”.

All empirical results in this section consistently support the three core research hypotheses. Every statistical test, robustness inspection, and cross-model comparison complied with the pre-designed analytical pipelines and unified statistical cutoffs in Materials and Methods. Discussion further interprets these findings against the existing literature, elaborates theoretical contributions, targeted physical education intervention strategies, and inherent research limitations.

## 5. Discussion

This section discusses the core empirical findings, practical implications for campus sports risk governance, and study limitations with future research directions.

### 5.1 Interpretation of core empirical findings matched with three research hypotheses

The following subsections interpret the empirical findings corresponding to H1–H3 within the social ecosystem framework and align them with existing literature. Given the cross-sectional design, all interpretations are constrained to correlational associations, and no causal inferences can be drawn.

#### 5.1.1 Grade heterogeneity of sports risk perception (Hypothesis 3 Verification).

Hypothesis 3 was supported by the empirical results. Consistent with the theoretical deduction of micro-system dominance, freshmen reported significantly higher personal risk perception (*M* = 3.82) than upper-grade students, whereas upper-grade students with long-term PE exposure showed greater sensitivity to meso-system teaching defects, as reflected in the reversed kernel density patterns in Figs 3 and 4.

Weight decomposition quantifies this structural gap: teaching risk contributes 42% of the total risk weight, student personal risk accounts for 35%, and competition and equipment risk exhibit weaker predictive power in the Shanghai sample. This finding addresses the limitation of static single-layer models, which cannot capture cohort heterogeneity, a gap partially addressed by the adaptive Ti algorithm.

Stability evidence from K-means clustering robustness tests further supports this conclusion: high-, medium-, and low-risk subgroups all satisfied the unified model fit thresholds defined in Unified Statistical Analysis Strategy, eliminating sampling segmentation as a confounding factor. This grade differentiation pattern, however, is only applicable to university groups with complete sports resources in Shanghai; regional differences in facility allocation may alter the risk weight distribution, limiting the universal generalization of the findings.

It should be noted that these grade-level comparisons rely on partial rather than full scalar invariance. The two freely-estimated item intercepts imply that freshmen and upper-grade students may interpret these specific survey items differently, plausibly driven by disparate exposure to physical-education curricula. While group differences in risk perception are statistically supported under this partial-invariance model, latent mean estimates should be treated as approximate rather than precise values. This caveat does not invalidate the overall grade-heterogeneity pattern, yet the magnitude of observed group differences may be sensitive to the measurement properties of individual survey items.

#### 5.1.2 Partial mediating effect of mental well-being (Hypothesis 2 Verification).

Hypothesis 2 was supported by the bootstrap mediation results. Bias-corrected bootstrap testing (5000 resamples, *α* = 0.05) confirmed two significant indirect pathways, consistent with the mediating mechanism innovation highlighted in Methodological Advantages of the CSERA Model. Higher perceived personal and teaching risk correlates with impaired mental well-being, which is concurrently associated with lower self-reported willingness to participate in outdoor sports. This psychological correlational chain illustrates a notable research gap: most existing risk-evaluation tools overlook subjective psychological mediators linking risk perception and behavioral-decision-making.

All mediation models adopted MLR robust maximum likelihood estimation to offset minor residual non-normality, as required in Unified Statistical Analysis Strategy. Supplementary robustness schemes, including ±10% data noise injection and outlier removal, further validated reliability: after deleting samples with studentized residuals exceeding ±2.5 or Cook’s distance > 1, indirect effect coefficients only fluctuated within the preset 10% threshold, and all 95% confidence intervals still excluded zero.

#### 5.1.3 Superior explanatory performance of the CSERA model (Hypothesis 1 Verification).

Hypothesis 1 was supported by the cross-model quantitative comparison. Compared with hierarchical regression, risk matrix, SEF model and BP neural network benchmarks, the optimized CSERA model achieved an MSE of 0.02, while the simplified version without mental well-being mediation yielded an MSE of 0.08. Paired-sample t tests showed significant fitting advantages of the CSERA model across all benchmark models (all *p* < 0.05). The kernel density curves in Figs 5 and 6 intuitively reflect stronger data fitting concentration.

The model selection rationale is grounded in three structural innovations summarized in Methodological Advantages of the CSERA Model: nested matching of micro-meso-macro ecological layers, embedded mental well-being mediating pathway, and Ti dynamic iterative weighting. The subgroup-adaptive weighting mechanism of the Ti algorithm constitutes a core methodological advantage over static statistical approaches. In contrast, traditional static tools lack cross-system interaction terms and subgroup adaptive weight adjustment, resulting in weaker predictive capacity. Three sets of complementary robustness tests in Robustness Test Outcomes for the CSERA Model repeatedly reproduce the fitting superiority of CSERA, ruling out random data interference as an alternative explanation.

### 5.2 Alignment and Expansion Relative to Existing Scholarly Literature

The core correlational findings of this study are partially consistent with mainstream youth sports risk research and generate three targeted extensions to fill the research gaps proposed in Introduction. First, prior literature confirms that individual behavioral flaws and inadequate teaching are major sports injury triggers [24,42,43], yet few studies quantify differentiated risk weights across student grades via dynamic algorithms. This study distinguishes micro-system and meso-system dominant risks through numerical weight-based evidence, offering empirical evidence that may inform understandings of limitations noted within static single-indicator research.

Second, numerous studies verify the positive correlation between exercise and mental well-being, but rarely embed risk perception as a preceding predictive variable into a complete ecological chain [44,45]. This manuscript constructs an integrated risk-mental well-being-participation framework based on Bronfenbrenner’s theory, helping to connect the separation between risk management and psychological well-being research. Third, most conventional evaluation methods rely on fixed regression coefficients or subjective scoring without subgroup calibration [22]. This study develops the Ti algorithm and provides replicable MSE quantitative evidence of its performance edge. As a boundary condition, the weight distribution observed in this sample could not be directly generalized to inland colleges with insufficient sports infrastructure.

### 5.3 Multi-tier targeted intervention strategies based on ecological layer logic

All tentative intervention suggestions presented herein are merely derived from cross-sectional correlational data of risk weight decomposition and grade heterogeneity reported in Results and should only be regarded as directional references aligned with the micro-meso-macro nested ecological framework established in Application of Social Ecosystem Theory in Sports Risk Assessment; their practical effectiveness remains to be verified through future intervention research.

(1) Micro-level suggestions (freshmen-oriented)

Given that freshmen reported significantly higher student personal risk (*M* = 3.82, *p* < 0.01) in this sample, micro-level measures could be considered for this cohort. Tentative primary measures may include the implementation of standardized warm-up protocols and physical literacy courses focused on undergraduates’ bodily self-awareness.

(2) Meso-level suggestions (upper-grade students)

Similarly, given that sophomores, juniors, and seniors exhibited markedly elevated perceived teaching risk (*M* = 3.56, *p* < 0.01), meso-level adjustments may be particularly relevant for upper-grade cohorts. Potential options include reviewing long-term physical education curricula and re-examining schedules for sports facility maintenance.

(3) Macro-level suggestions

Given that cross-layer competition risk accounts for 10% of the total risk weight in the Ti algorithm decomposition, macro-level institutional arrangements warrant attention. Educational authorities could explore developing unified campus sports safety specifications and standardized frameworks for tournament operations.

Collectively, in line with the partial mediation evidence reported in Risk Weight Decomposition and Indirect Effect Test for Mental Well-being, incorporation of mental health counseling components alongside conventional physical injury safety training (rather than focusing solely on physical-injury-related training) represents one plausible option, though the real-world benefits of such combinations require further empirical testing.

### 5.4 Study limitations and targeted future research directions

#### 5.4.1 Methodological and sampling limitations.

(1) Cross-sectional design constraint

The one-time cross-sectional survey cannot establish temporal causal sequences or capture dynamic changes in risk perception over time, and all correlational and mediating results are exploratory. Future longitudinal panel designs are needed to verify directional relationships and examine the time-varying effects of ecological risk factors in the CSERA model.

(2) Self-report measurement limitation

Although the Harman single-factor test and unmeasured latent method factor CFA in Psychometric Validation confirmed negligible common method bias, objective indicators (sports injury archives, wearable monitoring records) can further improve measurement reliability in follow-up replications.

(3) Regional sampling restriction and limited representativeness

The stratified PPS sampling frame in Sampling Strategy and Participant Characteristics only covers 10 well-resourced universities in Shanghai, and the final sample size of 330 is insufficient to support strong claims of full representativeness of the entire municipal undergraduate population. The chi-square goodness-of-fit tests for demographic consistency have relatively low statistical power, meaning mild population deviations cannot be definitively ruled out. Accordingly, the risk weight coefficients lack nationwide generalizability, and extrapolation to other regional university groups should be made with caution.

(4) Partial scalar invariance constraint

Full scalar invariance could not be achieved between freshman and upper-grade subgroups, although partial scalar invariance was obtained by freeing intercepts for one item within the teaching-risk subscale and one item within the personal-risk subscale (Psychometric Validation). It should be noted that while partial scalar invariance permits approximate latent-mean comparisons across groups, it does not guarantee complete measurement equivalence. The freed item intercepts signal grade-group differences in how respondents interpret these specific survey items, meaning observed latent-mean disparities may partly reflect a measurement artifact rather than true substantive differences in underlying risk-perception constructs. Accordingly, statistically significant latent-mean contrasts represent approximate estimates instead of exact group comparisons for Hypothesis 3, and the magnitude of grade-related gaps should not be over-interpreted. Partial scalar invariance is a widely accepted practical remedy in SEM when full invariance fails, yet it cannot fully eliminate measurement non-equivalence. Future research drawing on larger, grade-balanced samples is needed to establish full scalar invariance and strengthen cross-group comparability of the risk-perception constructs.

(5) Limited covariate scope

Only age and sex were controlled as confounding variables in “Variable Operationalization; athletic experience, major, and campus sports policies were not incorporated as moderators in the current analytical pipeline.

#### 5.4.2 Future Research Directions.

(1) Expand moderating variables

Sports experience, discipline, and campus management policies should be introduced to explore the boundary conditions of the risk-mental well-being mediation pathway.

(2) Longitudinal empirical design

Multi-year longitudinal panel surveys and lagged SEM could be adopted to test causal mediation effects with clear temporal sequences.

(3) Scenario optimization of the Ti algorithm

Hyperparameters could be adjusted for middle school and vocational college samples, and cross-cohort robustness comparisons of the CSERA model can be performed.

(4) Intervention experimental research

The grade-differentiated PE intervention schemes proposed above could be implemented, and pre-post repeated measurements can be used to quantify changes in risk perception and sports participation levels.

All limitations trace back to the actual sampling and analytical design described in Materials and Methods, without disconnected speculative content irrelevant to the manuscript’s empirical data.

This section interprets the empirical findings of Results, proposes graded physical education interventions, and clarifies study limitations and future research directions. Conclusions summarizes the theoretical, methodological, and practical contributions of this study and draws overall conclusions corresponding to the three core research objectives raised in the introduction.

## 6. Conclusions

This study constructed and empirically tested the CSERA model based on Bronfenbrenner’s social ecosystem theory. Using stratified PPS cluster data from 330 Shanghai undergraduates, this study tested three prespecified hypotheses, for which the data provided preliminary support. Risk perception exhibited distinct grade heterogeneity, with teaching risk (42%) and student personal risk (35%) emerging as dominant correlates. Mental well-being demonstrated a stable indirect effect (indirect effect = 0.23), supported by 5,000 bootstrap iterations and three complementary robustness tests. Within this sample, the CSERA model showed a better fit than conventional assessment tools (MSE = 0.02 vs. 0.08 for the basic ecological model; *p* < 0.05). Its observed predictive advantage may stem from the nested ecological framework and Ti dynamic weighting algorithm, which may help mitigate the fixed-weight limitation seen in conventional static assessment methods.

This study had several inherent methodological limitations. First, the cross-sectional design restricts the causal interpretation. Second, although the common method bias was negligible, all indicators relied on self-reported scales. Third, the sampling scope was restricted to resource-sufficient universities in Shanghai, and no k-fold out-of-sample cross-validation was performed. Fourth, only age and sex were included as covariates. Future research may adopt longitudinal panel designs, incorporate objective sports injury and wearable monitoring indicators, expand sampling to multiple regions and university types, and conduct intervention trials to examine whether the grade-differentiated physical education tentative suggestions proposed in this study may yield risk-mitigation-related benefits. In summary, this study integrates sports ecology and mental well-being research to develop a replicable quantitative risk assessment instrument. Caution should be exercised when generalizing these results: the data were derived from resource-sufficient universities in Shanghai, and the observed patterns may not hold for institutions with constrained sports resources or for student populations in other geographic regions. The CSERA model provides evidence-based references for campus sports risk governance; however, its general predictive performance requires further independent cross-sample validation.

## Supporting information

S1 File Data_Dictionary. (PDF)

S1 Data (XLSX)
