# Supplementary material for: Ecological insights and societal impacts: Unveiling the relationship between outdoor sports and mental well-being among college students
Source: PLoS One. 2026 Nov 10;21(11):e0360170. doi: 10.1371/journal.pone.0360170 (PMC13750570; doi:10.1371/journal.pone.0360170)
Supplement: Supplementary file 1 [file pone.0360170.s001.pdf]

Data Dictionary for the College Sports Risk and Mental Well-being Survey

File name: S1_Data.csv
Number of cases: 330
Data collection period: April 25, 2023 – October 25, 2023
Population: Undergraduate students from 10 universities in Shanghai, China
Inclusion criteria: Full-time students aged ≥18 years, with outdoor sports participation at least once per month

Variable List

Variable Name	Description	Coding / Range
ID	Anonymous participant identifier	1 – 330 (sequential)
Sex	Self-reported sex	1 = Male, 2 = Female
Major	Academic discipline category	1 = Liberal Arts, 2 = Science, 3 = Engineering
Grade	Year of study	1 = Freshman, 2 = Sophomore, 3 = Junior, 4 = Senior
Teaching_risk	Total score for teaching-related sports risk (e.g., insufficient warm-up instruction, inadequate supervision) .Number of items = 3 (each 1–5).	Sum ofLikert items (each item 1=strongly disagree to 5=strongly agree); theoretical range 3–15, observed range 7–15 (higher score = higher perceived risk)
Personal_risk	Total score for students' personal risk (e.g., overestimation of physical capacity, neglect of warm-up). Number of items = 3 (each 1 – 5).	Sum ofLikert items; theoretical range 3–15, observed range 6–15 (higher score = higher perceived risk)
Competition_risk	Total score for competition-related risk (e.g., pressure in formal competitions).Number of items = 3 (each 1 – 5).	Sum ofLikert items ;theoretical range 3–15, observed range 4–11 (higher score = higher perceived risk)

Variable Name	Description	Coding / Range
Equipment_risk	Total score for sports equipment risk (e.g., poor facility condition).Number of items = 3 (each 1 – 5).	Sum ofLikert items; theoretical range 3–15, observed range 3–10 (higher score = higher perceived risk)
Mental_well-being	Total score on the Warwick-Edinburgh Mental Well-being Scale (WEMWBS) – Chinese version	Sum of 14 items, each scored 1–5; theoretical range 14–70, observed range 40–61 (higher score = better mental well-being)
Outdoor_sports_frequency	Self-reported frequency of outdoor sports participation in the past month	Number of days per week; observed range 1–5
Outdoor_sports_duration	Typical duration per outdoor sports session	Minutes per session; observed range 20–120

Notes

All variables are complete (no missing values).

The dataset has been fully anonymized. No personally identifiable information (name, student ID, IP address, institution name) is included.

The number of items for each risk dimension is based on the original scales cited in the manuscript (Section 3.3.1) .

The survey items for each risk dimension are available from the corresponding author upon reasonable request, as they are adapted from previously published Chinese instruments (see manuscript Section 3.3.1 for original sources).

This dataset supports the findings reported in the manuscript “Ecological Insights and Societal Impacts: Unveiling the Relationship Between Outdoor Sports and Mental Well-being Among College Students”.
